# Value added medicines: what value repurposed medicines might bring to society?

**DOI:** 10.1080/20016689.2017.1264717

**Published:** 2016-12-23

**Authors:** Mondher Toumi, Cécile Rémuzat

**Affiliations:** ^a^ Faculté de Médecine, Laboratoire de Santé Publique, Aix-Marseille Université, Université de la Méditerranée, Marseille Cedex, France; ^b^ Pricing & Market Access Department, Creativ-Ceutical, Paris, France

**Keywords:** Value added medicines, drug repurposing, healthcare insufficiencies, sustainability, policy recommendations

## Abstract

**Background & objectives**: Despite the wide interest surrounding drug repurposing, no common terminology has been yet agreed for these products and their full potential value is not always recognised and rewarded, creating a disincentive for further development. The objectives of the present study were to assess from a wide perspective which value drug repurposing might bring to society, but also to identify key obstacles for adoption of these medicines and to discuss policy recommendations.

**Methods**: A preliminary comprehensive search was conducted to assess how the concept of drug repurposing was described in the literature. Following completion of the literature review, a primary research was conducted to get perspective of various stakeholders across EU member states on drug repurposing *(*healthcare professionals, regulatory authorities and Health Technology Assessment (HTA) bodies/payers, patients, and representatives of the pharmaceutical industry developing medicines in this field). *Ad hoc* literature review was performed to illustrate, when appropriate, statements of the various stakeholders.

**Results**: Various nomenclatures have been used to describe the concept of drug repurposing in the literature, with more or less broad definitions either based on outcomes, processes, or being a mix of both. In this context, Medicines for Europe (http://www.medicinesforeurope.com/value-added-medicines/) established one single terminology for these medicines, known as value added medicines, defined as ‘medicines based on known molecules that address healthcare needs and deliver relevant improvements for patients, healthcare professionals and/or payers’. Stakeholder interviews highlighted three main potential benefits for value added medicines: (1) to address a number of medicine-related healthcare inefficiencies related to irrational use of medicines, non-availability of appropriate treatment options, shortage of mature products, geographical inequity in medicine access; (2) to improve healthcare system efficiency; and (3) to contribute to sustainability of healthcare systems through economic advantages. Current HTA framework, generic stigma, and pricing rules, such as internal reference pricing or tendering processes in place in some countries, were reported as the current key hurdles preventing the full recognition of value added medicines’ benefits, discouraging manufacturers from bringing such products to the market.

**Discussion & conclusions**: There is currently a gap between increasing regulatory authority interest in capturing value added medicines’ benefits and the resistance of HTA bodies/payers, who tend to ignore this important segment of the pharmaceutical field. This situation calls for policy changes to foster appropriate incentives to enhance value recognition of value added medicines and deliver the expected benefit to society. Policy changes from HTA perspective should include: absence of any legislative barriers preventing companies from pursuing HTA; HTA requirements proportionate to potential reward; HTA decision-making framework taking into account the specific characteristics of value added medicines; eligibility for early HTA dialogues; Policy changes from pricing perspective should encompass: tenders/procurement policies allowing differentiation from generic medicines; eligibility for early entry agreement; non-systematic implementation of external and internal reference pricing policies; recognition of indication-specific pricing.

At the same time, the pharmaceutical industry should engage all the stakeholders (patients, healthcare providers, HTA bodies/payers) in early dialogues to identify their expectations and to ensure the developed value added medicines address their needs.

## Introduction

As a consequence of the 2008 economic crisis, a slowdown or even a decline in health spending growth has been seen in many Organisation for Economic Co-operation and Development (OECD) countries between 2009 and 2013 [1]. Health spending has been reported to have risen again since 2012, while growth remains below pre-crisis rates, especially in Europe [1,2]. Since then, European Union (EU) member states (MS) are exercising major pressure on health insurance budgets; various policies were implemented for rational use of medicines and to contain pharmaceutical expenditure, such as mandatory price cuts, changes in medicine co-payment rates, policies to promote use of generic and biosimilar medicines, increasing requirements in health technology assessments, and increasing restrictions in medicine access (reimbursement/prescribing restrictions) [3–5].

Healthcare system efficiency is a key challenge for policy-makers when countries have to ensure universal access and equity in patient access to health services while ensuring financial sustainability of their healthcare systems [6,7]. The imbalanced situation between increasing demand for access to better health services and healthcare products and budget constraints may challenge the sustainability of healthcare systems. It has been suggested that a non-linear relationship exists between healthcare expenditure and health outcomes, and that similar level of healthcare expenditure does not necessarily translate to similar health outcomes, suggesting some room to improve efficiency in many countries [6–8].

Drug repurposing is a concept known for many years and pertaining to the use of the current pool of existing molecules potentially re-positioned, re-formulated, or combined with new technological platforms and services [9]. Drug repurposing has the potential to provide new therapeutic alternatives for patients, delivering relevant clinical improvement while reducing clinical development time of molecules compared to *de novo* development of new chemical entity, with an economic advantage by decreasing medicine development times, but also through the optimisation of high quality affordable medicines such as generic medicines versus high expensive new molecules [10]. Value of drug repurposing is largely recognised by the scientific community, as seen with the current initiatives across the public, non-profit, and private sectors in this field [11]. For example, in 2012, the National Institutes of Health (NIH) National Center for Advancing Translational Sciences (NCATS) in the United States launched the ‘New Therapeutic Uses Program’, an initiative to develop partnerships between pharmaceutical companies and academic scientists in drug repurposing; pharmaceutical companies agreed to provide access to their partially developed therapeutic candidates (referred to as *assets*) and related data [12]. Moreover, drug repurposing is a topic of high interest for European regulators, and was put at the agenda of European Commission Expert Group on Safe and Timely Access to Medicines for Patients (‘STAMP’) in March 2016 [13].

Despite the wide interest surrounding drug repurposing, no common terminology has been agreed for these products, and their full potential value is not always recognised and rewarded, creating a disincentive for further development.

The objectives of the present study were to assess from a wide perspective (healthcare providers, patients, regulators, and payers) which value drug repurposing might bring to society, but also to identify key obstacles for adoption of these medicines and to discuss policy recommendations.

## Methodology

A preliminary comprehensive search was conducted to assess how the concept of drug repurposing was described in the literature. The search was conducted in English language, limited to publications released between 2000 and December 2015 (cut-off date of the search) in the following databases: Medline; Embase; Cochrane; Generics and Biosimilars initiative (GaBi) website. The search strategy included preliminary free search terms (‘marginal innovation’, ‘incremental innovation’, ‘adaptive innovation’, ‘re-innovation’, ‘hybrid products’, ‘drug repurposing’, ‘drug reformulation’, ‘drug repositioning’), and was complemented with additional free search terms found in the literature.

Following completion of the literature review, a primary research related to drug repurposing was conducted across EU MS to define from key stakeholders’ perspectives (patients, healthcare professionals, regulatory authorities, and Health Technology Assessment (HTA) bodies/payers): (1) how drug repurposing was currently perceived; (2) how drug repurposing might respond to their needs; and (3) which actions might be undertaken to enhance value recognition and access of drug repurposing across Europe. Eight examples of repurposed medicines were presented to them to get their perspectives (Table 1). One-hour telephone interviews were conducted with 20 stakeholders among healthcare professionals, regulatory authorities, and HTA bodies/payers (profiles presented in Table 2) using a standardised discussion guide (see Supplementary file 1). The discussion guide was structured around five sections: healthcare system inefficiencies perception; knowledge of drug repurposing concept; reaction to different profiles of repurposed medicines; perspective on current recognition of drug repurposing; and perspective to enhance recognition of drug repurposing. During the first European Patients’ Forum – Medicines for Europe Dialogue, which took place on 31 May 2016, the patient’s perspective was collected, following presentation of key examples of repurposed medicines to assess their perception of these medicines and how these medicines might respond to their needs. (see Supplementary file 2).

**Table 1.** Example of cases of repurposed medicines to assess stakeholders’ perception.

Case 1	A fixed-dose combination of two products already available on the market and used as free dose combination in arterial hypertension to reduce pill burden and avoid intake errors in a highly medicated patient population.
Case 2	A self-injected subcutaneous formulation of a product already available on the market as an intravenous formulation administered only at hospital under medical monitoring in a severe inflammatory disease.
Case 3	A new formulation of a well-known chemotherapy product helping to reduce serious side effects of the original product used in many chemotherapy regimens.
Case 4	Re-positioning of a well-known product in a rare pediatric indication as an alternative to reference treatments not specifically approved in this indication.
Case 5	A new inhaled device to administer genericised products in COPD indication, with evidence of reducing inhaler errors versus current device used with these active substances.
Case 6	An extended-release formulation of a product already available on the market, reducing administration regimen from once-weekly injection to three-monthly injection in a neurocognitive disease indication.
Case 7	A therapeutic drug monitoring device in association with a known cancer therapy exhibiting a narrow therapeutic window to potentialise drug efficacy while minimizing toxicity.
Case 8	An injectable biological to be kept refrigerated that will be provided to the patients with cool bags and sharp containers (not provided with the reference product), aiming to facilitate daily usage by the patients.

**Table 2.** Primary research with healthcare providers/payers/HTA bodies/regulatory authorities; Stakeholders’ profiles.

Country	Perspective	Education	Organisation(s)
Austria	HTA body’s representative	Communications & psychology, PhD in social medicine	HTA body: Ludwig Boltzmann Institute for Health Technology Assessment (LBI-HTA), Vienna (academic non-profit institute, independent entity for scientific decision-making support in the health sector)
Belgium	Health insurer	PharmD	National Health Insurance: National Institute for Health and Disability Insurance (NIHDI RIZIV/INAMI)
France	Hospital pharmacist	PharmD	Public teaching hospital in Paris/hospital pharmacy
France	Physician	MD, PhD Specialty: psychiatry	Public teaching hospital in Marseille
France	HTA body representative (Ex)/physician	MD, general practitioner	Transparency Committee (French HTA, committee within French Health Authority, HAS) Private practice as GP
France/ Europe	HTA body’s representative/regulatory authority’s representative/physician	MD Specialty: dermatology/ endocrinology	Haute Autorité de Santé (HAS) European Network for Health Technology Assessment (EUnetHTA) French Medicines Agency (AFSSAPS/ANSM) European Medicines Agency EMA)
France/ Europe	Regulator (Ex)/physician	MD Specialty: cardiology/ diabetology (hospital)	European Medicines Agency (EMA/CHMP) Teaching hospital in Paris
Germany	Health insurer/proxy payer	MD (epidemiologist)	Public Physicians Association (Association of Physicians of Hessen) G-BA (Federal Joint Committee) advisor
Germany	Proxy payer (expert/advisor)	Health economist, PhD in public health	German Social Health Insurance
Italy	Physician	MD Specialty: neurologist	Public hospital in Cesena
Italy	HTA body’s representative/hospital pharmacist	PharmD	HTA body in Piedmont Region Public hospital in Turin
Italy	HTA body’s representative/hospital pharmacist	PharmD	HTA body in Sicily region Public hospital in Messina
Poland	Hospital pharmacist	PharmD	Public teaching hospital in Krakow
Poland	Physician	MD, PhD	Out-patient specialist care unit (commercial): ENEL-MED, Cracow, Poland; UNICARDIA, Cracow, Poland
Poland	Ex-HTA body’s representative	MD, PhD	HTA body: The Agency for Health Technology Assessment and Tariff System (AOTMiT), Warsaw
Spain	HTA body’s advisor	Health economist	HTA regional bodies (incl. Catalonia, Basque Country, Andalusia, Galicia)
The Netherlands	HTA body’s representative (ex/advisor)	Econometrics, health economist, PhD	HTA body: CVZ (now called ZIN (Zorginstituut Nederland))
Sweden	HTA body representative (ex/decision-maker)	Health Economist, PhD	HTA body: Dental and Pharmaceutical Benefits Agency (TLV)
Sweden	Proxy payer/HTA body (academic)	Health economist, PhD	Public University, Department of Medical and Health Sciences, Division: Centre for Medical Technology Assessment, Linköping University
United Kingdom (Scotland)	HTA body’s advisor	Health economist	HTA body: Scottish Medicines Consortium

Finally, a written survey (see Supplementary file 3) complemented by a focus group was also conducted among representatives of the pharmaceutical industry developing medicines in this field to assess their perspective of market access of repurposed medicines.

An *a* *d hoc* literature review was conducted to illustrate, when appropriate, statements of the various stakeholders.

Results of the primary and secondary research were then compiled together to present the potential contribution of repurposed medicines to healthcare systems and barriers to recognition of their value from a broad perspective.

## Results

### Drug repurposing terminology

Various nomenclatures have been used to describe the concept of drug repurposing in the literature, with more or less broad definitions based on outcomes, processes, or a mix of both (Table 3). In this context, Medicines for Europe (http://www.medicinesforeurope.com/value-added-medicines/) established one single terminology for these medicines, known as value added medicines, defined as ‘medicines based on known molecules that address healthcare needs and deliver relevant improvements for patients, healthcare professionals and/or payers’, and based on three repurposing models: drug repositioning; drug reformulation; and drug combination (drug/drug or drug/device or drug/service). Relevant improvements delivered by value added medicines include a better efficacy, safety and/or tolerability profile, a better way of administration and/or ease of use, and new therapeutic uses (in terms of indication or population), and those improvements may contribute to improve adherence, health outcomes or quality of life, improve safety and efficiency of healthcare professional resources, increase treatment options and prevent therapeutic escalation, and improve cost-effectiveness and ultimately access to healthcare [14].

**Table 3.** Nomenclatures and definitions related to drug repurposing.

Nomenclature	Definition(s)
Super generics	‘Improved version of an original drug which has lost product patent protection’. [15] Also referred to as added value generics, generic plus, innovative generics. There is a reported shift in this terminology for ‘non-generic identity’ (hybrid terminology most commonly used). [16]
Premium generics	Nomenclature defined by Daiichi Sankyo Espha (Daiichi Sankyo generics subsidiary) with the launch of ‘high value-added generic drugs’, including ‘innovations in formulation and labelling to make drugs easier to ingest and harder for patients to mistakenly or incorrectly take’. [17]
Specialty generics	Generic drugs which ‘benefit from more sophisticated technologies (such as controlled or immediate release) or from special pharmaceutical ingredients (self-molecules or biological active substances)’ [18]
Re-innovated generics	‘Products built upon a re-innovation framework between incremental and radical innovation. They improve the next generation with revised and refined features’. [15]
New therapeutic entities (NTEs)	Nomenclature defined by TEVA as ‘new specialty medicines based on known and approved chemical molecules. These molecules are reformulated, repurposed or re-engineered to be delivered in a new way to address specific, unmet patient needs.’ [19]
Enhanced therapeutics	‘Drug products derived from existing generic drugs that provide additional benefits to the patients and the healthcare system.’ [20]
Improved therapeutic entities	‘Products that offer a therapeutic advantage or differ from the me-too generic product in the sense of a patient centric drug delivery or product design or simply a more efficient product design and manufacturing process.’ [21]
Incremental innovation	‘Closely related molecules with different attributes that may offer significant value in treating particular disease variants or patient segments.’ [22] ‘Process of exploring and improving radical products.’ ‘Improvements in therapeutic quality, safety, and efficacy over existing medicines.’ [23] ‘Creating minor improvements or simple adjustments in a product’s current state.’ [15] ‘Either new approved drugs created from an already existing molecule or approved modifications to existing drugs.’ There are five types of incremental innovation [24]: new dosage form: which affects the dosage route, the dosage form and the does amount; new formulation: how the chemicals in the drug are combined to produce the drug; new combination: creation of combination drugs from existing molecules; new indication: using an existing drug to treat a different condition; new active ingredient: drugs that contain the same active moiety but include a different enantiomer, racemate, salt, ester, complex, chelate, or clathrate.Also referred to as adaptive innovation [23] or marginal innovation. [25,26]
Re-innovation	‘Process of innovation and product development that occurs after a new product is launched, building upon early success but improving the next generation with revised and refined features.’ ‘Re-innovation by the generic pharmaceutical industry can be observed in drug product design, formulation, process development and manufacturing processes going back to the early stages of the product development cycle.’ ‘The re-innovative product (as compared to an incremental new product) can be defined as a product that provides new features, benefits, or improvements through existing technology.’ ‘This kind of value-added version is manufactured in a re-innovation framework. This innovative design is between incremental and radical innovation.’ [15]
Hybrid products	‘In cases where the medicinal product does not fall within the definition of a generic medicinal product as provided in paragraph 2(b) or where the bioequivalence cannot be demonstrated through bioavailability studies or in case of changes in the active substance(s), therapeutic indications, strength, pharmaceutical form or route of administration, vis-à-vis the reference medicinal product, the results of the appropriate pre-clinical tests or clinical trials shall be provided (as per Art. 10(3) of Directive 2001/83/EC).’ [27,28]
Bio-superior products	‘Intended to have attributes that are better than the first-generation product (…) A bio-superior utilises cutting-edge technologies such as protein engineering, and novel drug formulation and delivery approaches to enable its superiority over a first-generation product, possibly improving its efficacy or safety profile or improving administration route or reducing dosing frequency.’ [29]
‘Third Sector’ drugs	‘Compared to a NCE (New Chemical Entity), a Third Sector brand uses a proven molecule, lowering time and costs in development and, depending on the innovation, reducing regulatory risk. Compared to a generic, a Third Sector brand has a certain level of differentiation by addressing a specific payer, healthcare provider or patient unmet need and can aim for a higher price and/or market share. Some Third Sector brands may also have exclusivity and patent protection of some element of the offering, for example, a unique delivery system which generics cannot copy.’ [30]
Drug repurposing	‘Includes all the re-development strategies based on the same chemical structure of the therapeutically active ingredient as in the original product.’ [9]
Drug reformulation	‘Reformulation is, by the simple definition of the term, making a particular change in the formulation of the original drug. This can be achieved by exploiting advances in formulation technology to change the release of the active substance, pharmaceutical forms, and/or route of administration but it can also concern some excipients with no impact on the pharmacokinetic parameters. No change should be incurred in the structure of the active pharmaceutical ingredient except when it is a chiral switch. … Cases where the development of a new product does not include a change in the original formulation (i.e., change of dose, package size, etc.) should also be excluded.’ [9]
Drug repositioning	‘Process of finding a new indication for a drug or compound. … New indication is distinct from the already approved/intended indication of the original product, where “distinct” implies an anatomical and/or therapeutically distinct indication referring to the 10th version of the International Classification of Diseases (ICD-10). The situation where the new indication involves a different pharmacological target (off-target repositioning) is the only exception where a new use in a similar indication will be covered by the actual definition.’ [9]
Drug re-profiling/ Drug reusing/ Drug rediscovery	‘The usage of known drugs for new diseases. The main objective of drug re-profiling is to discover methods for using approved drugs or discarded clinical candidates in the treatment of new diseases.’ [31]

Value added medicines terminology was then used for primary search and is to referred as such for the next sections of the manuscript.

### Potential contribution of value added medicines to healthcare systems

Stakeholder interviews highlighted three main potential benefits for value added medicines: (1) to address some healthcare system inefficiencies related to medicines; (2) to improve healthcare system efficiency; and (3) to contribute to sustainability of healthcare systems through economic advantages.

#### Value added medicines may represent an opportunity to address some healthcare system inefficiencies

Stakeholder interviews supported by *ad hoc* literature review reported various healthcare system inefficiencies related to medicines that might be addressed by value added medicines: (1) irrational use of medicines; (2) non-availability of appropriate treatment options; (3) shortage of mature products; and (4) geographical inequity in medicine access.

##### Irrational use of medicines

The World Health Organization (WHO) considers irrational use of medicines wasteful and harmful for both the individual and the population [32]; this can contribute to increase the risk of adverse medicine events and lead to morbidity, hospitalisation, and mortality. For example, irrational use of antibiotics (overuse, inappropriate choice, poor adherence) is a key public health issue leading to development of antimicrobial resistance, causing about 25,000 deaths per year in the EU and resulting in extra healthcare costs and productivity losses of at least €1.5 billion each year [33–35].

Irrational use of medicines can take different forms. First, poor treatment adherence is reported as a major barrier to achieving the potential benefit of available medicines; an overview of adherence to long-term therapies conducted by the WHO in 2003 found around 50% adherence as the average rate in developed countries [36]. Poor adherence has been estimated to cost about €125 billion annually to European governments, and to contribute to the premature deaths of nearly 200,000 Europeans annually [37]. Second, off-label use of medicines in indications with little or no evidence supporting use, and when alternative approved effective therapies do not exist, is frequent and particularly high in some specific therapeutic areas, such as oncology (in a position paper of the European Society for Medical Oncology [ESMO], off-label use of oncology medicines was estimated to reach approximately 50% [and even more] [38]), and in certain patient groups, especially in paediatrics (estimated between 33.2% and 46.5% in inpatients and between 3.3% and 13.5% in out-patients, despite European paediatric regulation [2007] [39]). Finally, irrational use of medicines also encompasses polypharmacy when the use of multiple medicines is not medically necessary, the lack of treatment coordination (e.g., duplication of prescriptions), non-conformance with prescribing guidelines, prescribing inefficiency, with variation in the use of medicines between physicians and underuse of generic and biosimilar medicines, and medicine wastage (e.g., vial wastage with inappropriate volume size or tablet wastage with inappropriate pack size).

By optimising treatment regimens, value added medicines could contribute to improve rational use of medicines. Through new drug formulations or drug combinations, value added medicines could contribute to improve adherence issues of already available therapies. They could also contribute to improve patient convenience of use and satisfaction with healthcare through improvement of their usual therapies, which might further contribute to enhancing patient adherence, especially for patients treated for chronic diseases. Notably, the WHO recognises the importance of value added medicines in improving patients’ adherence and the fight against resistance to antimicrobials. For instance, in patients with both tuberculosis and HIV infection, the WHO supports innovations, such as the use of fixed-dose formulations of multiple antimicrobial components, to encourage patients’ compliance with treatment, thus limiting the risk of resistance [34]. Through drug repositioning and drug reformulations for specific patient groups (e.g., paediatric population), value added medicines could also contribute to limit off-label use of medicines. Moreover, through new and appropriate medicine packaging and vial conditioning, value added medicines could contribute to limit medicine wastage.

To illustrate the optimisation of treatment regimen, we could cite two examples of current approved value added medicines: (1) DuoResp Spiromax® (budesonide/formoterol) is a combination of already existing molecules associated with a new inhaler, Spiromax®,designed to improve ease of use, consistent dose delivery, and confirming dose intake to patients through a taste of lactose and a dose indicator; (2) the first liposomal formulation of doxorubicin, Doxil®, was developed to reduce toxic effects of doxorubicin.

##### Inappropriate treatment options

Some therapeutic areas are facing a decline in development of innovative approaches. For example, in mental health, a decline of innovation has been reported, with decreasing investments in research and development in new treatments for depression, bipolar disorder, schizophrenia, and other psychiatric disorders [40–42].

In the field of infectious disease, there is a high demand for new generations of antibiotics in the current context of antibiotic resistance. Only five new classes of antibiotics have been launched since 2000, and high unmet needs remain for new molecules targeting especially gram-negative bacteria [43,44].

On top of this, current therapies are not well tailored to meet the particular needs of different patient sub-groups, such as vulnerable patients (e.g., pregnant woman, elderly patients, and paediatric population), or patients requiring frequent dosing adjustments, which may lead to inadequate clinical practice to adjust available therapies to patient medical needs, including off-label use.

Value added medicines may represent an opportunity to tailor and expand access of well-known therapies to particular patient subgroups’ needs, such as vulnerable patients or patients requiring frequent dosing adjustments. They could contribute to the faster development of new therapeutic options in areas of unmet medical needs, benefiting from the knowledge gained from the previous medicine development and life cycle. It may also happen through the evolution of scientific knowledge, for example, when a new mode of action is discovered for an existing product or a new effect is discovered for a well-established mode of action.

For example, Pheburane® (sodium phenylbutyrate) is a repurposed formulation of Ammonaps®, developed as a coated granule formulation that reduces/removes the bitter taste associated with the active substance (different excipient used to mask the unpleasant taste of the active substance) designed to improve treatment compliance, particularly in children.

##### Shortage of mature products

Lack of financial attractiveness and the ability to competitively supply the market, for example, through single lot tenders, or lack of cost coverage to maintain the marketing authorisation and supplying of some older essential medicines may result in in stock-outs or market withdrawals by manufacturers. For example, this was the potential reason for withdrawal of extencilline from the French market in 2014, the sole antibiotic for the treatment of syphilis available in the territory and which now requires medicine importation from Italy [45,46].

Value added medicines may provide an opportunity to create new market attractiveness of mature products, which could avoid product shortage in some countries.

##### Geographical inequity in medicine access

Disparities in medicine access are seen between European countries that may not only be driven by affordability, although it is a critical driver of poor access. For example, a study assessing access to oncology care in four European countries (France, Germany, Sweden, and Poland), based on a review of colorectal, lung and prostate cancer care, found inequalities in access to cancer medicines. Highest and quickest uptakes were seen in France; Germany and Sweden showed similar uptakes for established medicines, but Sweden had lower uptakes for new cancer medicines; Poland was far behind these countries, with no uptake of some newer cancer medicines [47].

Moreover, these disparities in medicine access are also found within countries, especially when pharmaceutical budgets are managed at regional levels. For example, in Italy, a survey conducted by the Italian Society of Medical Oncology (AIOM) in 2009 showed disparities in oncology medicine access between Italian region in terms of inclusion in the regional pharmaceutical formularies and time to patient access [48].

Value added medicines might contribute to addressing geographical inequities, either by the opportunity they represent to create an intermediate step before switching to costly products, thus improving the affordability and limiting geographical access inequity, or by the opportunity they represent to provide new drug formulations for hospital-only medicines, which could be used in out-patient settings, thus improving access in remote rural areas, for example.

#### Value added medicines may represent an opportunity to improve healthcare system efficiency

Value added medicines may represent an opportunity to better address healthcare provision and organisation and could contribute to a reduction and re-allocation in healthcare use. For example, chemotherapy reconstitution process is not related to healthcare system inefficiencies; however, a ready–to-use chemotherapy which might improve medicine handling and save time for healthcare providers would represent an important benefit in terms of use of available healthcare resources.

#### Value added medicines may contribute to favourably impact healthcare budgets

**Figure 1.**
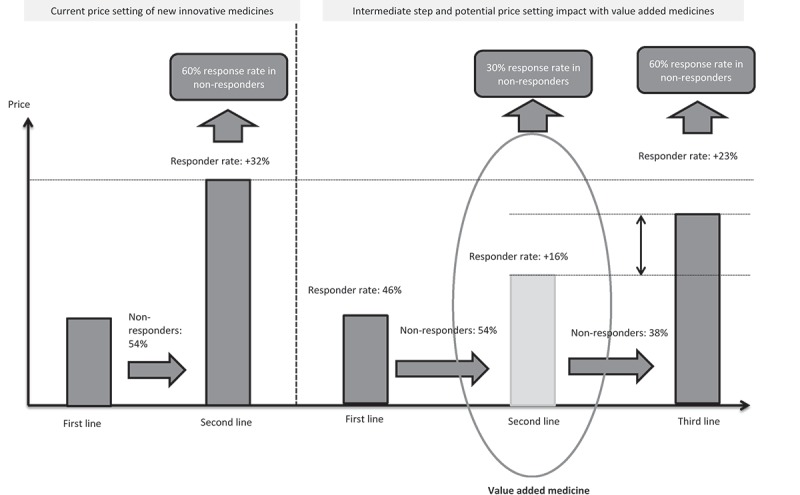
Illustrative representation of intermediate step created by value added medicines and potential price.

In the current cost-constrained environment, value added medicines may represent an opportunity to create an intermediate step before switching to costly products, as well as to reducing budget impact. Value added medicines may have a potential impact on price-setting and budget if an expensive innovative medicine (with improved efficacy profile versus value added medicines) is expected to be launched in the same indication. For example, in some cases, value added medicines might be pushed as a second-line option versus the well-established product, while most innovative therapy might be niched as third-line option, after the value added medicine step, which could have a positive budget impact (Figure 1).

### Key obstacles for the adoption of value added medicines

Pricing and reimbursement pathways of value added medicines were reported as the current key hurdles preventing an optimal utilisation, or even preventing the development and market launch of value added medicines. One significant obstacle identified to obtaining benefit acknowledgement of value added medicines during pricing and reimbursement process was the stigma surrounding these products, which may be alternatively perceived by stakeholders like generic medicines, or as an anti-generic medicines strategy preventing from capturing any savings from medicine falling off patent.

#### Health technology assessment and medicine coverage-related issues

##### HTA decision-making framework

###### Complexity to evidence the benefit of some value added medicines

In the current cost-constrained environment, there is an increasing demand for robust evidence to demonstrate the additional benefit of a new medicine versus the therapeutic strategy, with a growing request for real-world data. In some cases, the evidence of the benefit of value added medicines may be complex to be demonstrated when evidence relies on improvement of patients’ preference, compliance, convenience of use, surrogate endpoints, etc. Such benefits are poorly captured, if at all, by Quality-Adjusted Life Year (QALY), which is the reference measure of medicine value in several countries, and require substantial investments to be proven through study designs acceptable by HTA agencies. However, the level of requested evidence is generally disconnected from relevant reward from HTA bodies and ultimately by payers.

###### Separate assessment of medicines and devices/procedures in some countries

HTA of medicines and devices or procedures are performed separately in some European countries, which prevents HTA from fully capturing the benefit of some value added medicines using a medicine and device or procedure combined, and can lead to patient access delays or even inconsistent decisions when processes are not coordinated. For example, pharmaceutical medicines and the associated companion diagnostics are evaluated separately in France, Germany, Italy, and Spain, while in England companion diagnostic evaluation is integrated into the National Institute for Health and Care Excellence (NICE) technology appraisal of the associated medicine [49].

###### Different HTA and medicine coverage procedures between medicine classes

Some European countries implemented different HTA and medicine coverage procedures between medicine classes. For example, orphan or end of life medicines can enjoy privileged assessments. For example, in 2009, the NICE introduced end of life criteria to improve access to end of life treatments which could potentially be recommended at a higher cost-effectiveness threshold than ‘standard’ medicines. In 2014, the Scottish Medicines Consortium (SMC) introduced a new process for assessing medicines treating end of life and very rare conditions (orphan and ultra-orphan medicines), setting up a Patient and Clinician Engagement (PACE) group to give patient groups and clinicians a stronger voice in the SMC decision-making process. Another example relates to conditional reimbursement that can be restricted to specific categories of medicines; for example, expensive hospital-only medicines in the Netherlands [50]. In some European countries, HTA is not performed for all medicines, i.e., categorised as generic medicines or for hospital-only medicines. For example, in Germany, hospital-only medicines are exempted from early benefit assessment procedure. As such, depending on countries and product category, value added medicines might not be eligible for HTA and might be grouped with generic medicines for reference pricing.

##### Budget silos

Some European countries tend to consider pharmaceutical assessments and reimbursement decisions in a silo, preventing the capturing of any benefits such as transfer of cost-savings outside of the pharmaceutical expenditure budget. For example, in Belgium, the reimbursement decision might not consider any savings that may result from a new medicine that would decrease the number of hospitalisations. Within healthcare budgets, previous work analysing medicine reimbursement policies in Europe reported a ‘drug budget silo’ mentality likely to lead to inefficiency when pharmaceutical expenditure is considered separately to overall healthcare resource budget [51]. On top of this separation between pharmaceutical and other healthcare resources, a trend has been reported of separating healthcare budgets from other related budgets, such as social care [52]. As such, value added medicines’ benefits may not be fully captured by HTA bodies due to a budget silo, for example, by cost-savings achieved across a hospital healthcare organisation.

#### Medicine pricing rules-related issues

##### Pricing policies pushing price down

By pushing price down, some pricing policies might negatively impact value added medicines access, such as: (1) systematic positioning as generic medicine and inclusion of value added medicines in internal reference pricing groups based on active substance; (2) tenders/procurement policies with award criteria based exclusively on economic criteria for active substance (lowest price); (3) external reference pricing, especially when value added medicines are considered differently from a pricing and reimbursement perspective (e.g., internal reference pricing, tendering, etc.).

Moreover, some payers may integrate investment risk in their decision with a preconceived opinion that the investment risk in originator product is higher, so the value should be weighted by the risk to set the price. There is a clear feeling from payers that a repurposed product is an obvious low risk investment; therefore, their willingness to pay may be lower for the same added value as a new therapeutic class product. This is seen in France, for example, where the Pricing Committee (CEPS) takes into account the level of risk taken by the company when setting prices [53].

##### Issues related to differential pricing across indications

Many medicines are currently approved for multiple indications, with potential different value across indications. European countries generally apply a single price across all indications; however, some countries achieved indication-specific pricing through different mechanisms. For example, in France, HTA is conducted per indication, and the price is based (among other criteria) on a weighted average of the value of all the indications. In Italy, different payback schemes for the same medicine are agreed per indication between the Italian Medicines Agency (AIFA) and the medicine manufacturer at the time of reimbursement decisions.

Uncertainty surrounding differential pricing across indications may either restrict access to the most cost-effective indications if the price is based on the indications with the highest value, or disincentive companies from launching the medicine in indications with the lowest value, thus depriving society of the treatment needed to address an unmet need.

## Discussion

There is currently a gap between increasing regulatory authority interest in capturing value added medicines’ benefits and the resistance of HTA bodies/payers, who tend to ignore this important segment of the pharmaceutical field.

Indeed, recent initiatives demonstrated the importance of drug repurposing from a public health perspective and the willingness of regulatory stakeholders and public health authorities to encourage their development. ‘Repurposing of established medicines’ is currently in discussion at the European Commission, with the Commission Expert Group on Safe and Timely Access to Medicines for Patients (‘STAMP’) recognising the importance of fully investigating different opportunities that a molecule could bring for patients, with faster development times, at reduced costs and risk for pharmaceutical companies; the group will consider the opportunity to provide a standardised definition [13]. The background note prepared by the UK Medicines and Healthcare products Regulatory Agency (MHRA) to provide a basis for STAMP’s consideration of the issue cites current European regulatory incentives for drug repurposing, which include [13]: a non-cumulative period of one year of data exclusivity granted for a new therapeutic indication for a well-established substance, provided that significant pre-clinical or clinical studies were carried out in relation to the new indication (Paragraph 5 of Article 10 of Directive 2001/83/EC); a period of data and market protection of 8 + 2 years covering indication(s) and appropriate formulation(s) for already authorised products developed for paediatric populations (Paediatric-use Marketing Authorisations (PUMA), Article 30 of Regulation (EC) No 1901/2006); a market exclusivity of 10 years for repurposed medicines granted an orphan drug designation.

In addition, as authorities are aware of the disincentive for manufacturers to repurpose mature products and to invest in this field, they have launched a number of initiatives to enhance such practice in order to secure opportunities to identify and capture their whole benefit. For example, new partnerships have been established between public funders, pharmaceutical industries, and academic investigators in drug repurposing (e.g., the Medical Research Council in the United Kingdom partnered with AstraZeneca in 2011 to give access to clinical and preclinical compounds to academic researchers for potential repurposing [54]; in France, the National Cancer Institute (INCa), in agreement with the French National Agency for Medicines and Health Products Safety (ANSM), launched in 2013 the AcSé (Accès sécurisé à des thérapies ciblées innovantes) programme which is a programme for secure access to innovative targeted therapies, offering cancer patients, for whom validated therapies have failed, access to targeted therapies, based on a molecular abnormality of their tumour; the AcSé crizotinib project was the first clinical trial of the AcSé program [55]); these programmes aim to identify potential value in products that were either shelved or already on the market for a more or less long period, and whether or not covered by a patent.

Finally, some national initiatives already regulate the healthcare inefficiency related to off-label use of marketed medicines use, such as Temporary Recommendation for Use (RTUs) in France [56]; RTU was implemented to allow safer off-label use of medicines in situations of unmet therapeutic need, and to ensure an adequate patient monitoring is setting up by the pharmaceutical company, to finally improve knowledge in the new indication and encourage the company to request an extension of the marketing authorisation in this indication.

These initiatives highlight the real need to enhance the recognition of value added medicines by all stakeholders as medicines offering societal value distinct from originator and generic products based on the same active substance.

Moreover, the global perception of the lack of reward for value added medicines by manufacturers might lead them not to invest in such products, not to launch, or to withdraw value added medicines from some less favourable countries, leading to inequities in value added medicines patient access across countries.

This situation calls for policy changes to foster appropriate incentives to enhance value recognition of value added medicines from HTA and pricing perspectives and deliver the expected benefit to society.

In terms of pricing and reimbursement pathways, policy change should involve getting pricing and reimbursement rules that offer the possibility for HTA pathways to take into account specific characteristics of value added medicines that should not be assimilated systematically to generic medicines because of the lack of new chemical entity. First, there should be no legislative barriers preventing companies from pursuing HTA for selected value added medicines in order to demonstrate relevant improvements for patients, healthcare professionals, and/or payers. When HTA is performed, HTA requirements should be proportionate to potential reward (i.e., if a considerable amount of money is invested to fit with HTA requirements, this should be rewarded). For example, if a potential value added medicines benefit is rated as modest by HTA bodies, regardless of outcome, a very complex, heavy, long, and expensive study should not be required; ultimately, the possible expected economic reward should be proportionate to the level of requirements from HTA bodies. Moreover, the HTA decision-making framework should take into account the specific characteristics of value added medicines not currently captured; i.e., to enlarge the scope of benefits of value added medicines: for example, include patients’ and healthcare providers’ preferences, but also give more weight to quality of life and health economic benefit; and to accommodate different time points at which evidence can be assessed – for example, the use of modelling techniques to predict the outcome, as well as coverage with evidence development to capture real world benefits. Of note, value added medicines should be eligible for multi-HTA early dialogue and parallel scientific advice (EMA-Multi-HTA early dialogue) to support, at best, further medicine development plan while the process is targeting mostly innovative originator medicines.

Another important policy change would imply enforcing pricing policies rewarding value added medicines development as for new chemical entity: i.e., (1) value added medicines specificities should be acknowledged in tenders/procurement policies to allow differentiation from generic medicines; (2) early entry agreement should be made available for value added medicines to allow bringing evidence along commercialisation; 3) external and internal reference pricing should not apply systematically for value added medicines. Moreover, indication-specific pricing should be allowed for medicines having multiple indications, with potential different value across indications.

On another level, the pharmaceutical industry should adopt a proactive approach when willing to develop and market value added medicines. The industry should engage in early dialogues with HTA bodies/payers to best fit their expectations for value added medicines’ development and obtain recognition of additional value. They should also involve patients’ groups and healthcare providers in identifying their needs and ensuring that the developed value added medicines address established and well-documented unmet needs.

At the same time, companies should raise acceptance of value added medicines through communication campaigns, differentiating value added medicines from generic medicines, and decrease stigma as counter-acting generic medicine perception by demonstrating their value on all the features described previously.

During the clinical development process, manufacturers should validate surrogate endpoints. For example, if a company is developing a specific technology, such as a specific new inhaler developed for administration of medicines to treat asthma, one study might be conducted to validate that the reduction of error with inhaler X is correlated with a better efficacy in terms of reduction in asthma exacerbation; for all products developed with inhaler X, the evidence in the reduction of errors with inhaler X might be considered as valuable to acknowledge the benefit in terms of efficacy.

Finally, manufacturers should invest in patient registries and post-authorisation studies to collect real world data.

## Conclusion

Value added medicines represent an opportunity for society to address a number of medicine-related healthcare inefficiencies related to irrational use of medicines, non-availability of appropriate treatment options, shortage of mature products, geographical inequity in medicine access, and also present an opportunity to deliver better health to patients, to enhance healthcare system efficiency, and contribute to the sustainability of healthcare systems.

There is currently a gap between increasing regulatory authority interest in capturing value added medicines’ benefits and the resistance of HTA bodies/payers, who tend to ignore this important segment of the pharmaceutical field. Current HTA framework, generic stigma, and pricing rules, such as internal reference pricing or tendering processes in place in some countries, prevent the full recognition of value added medicines’ benefits, discouraging manufacturers from bringing such products to the market.

This situation calls for policy changes to foster appropriate incentives to enhance value recognition of value added medicines from an HTA and pricing perspective and deliver the expected benefit to society.

## Supplementary Material

Supplementary file 3

Supplementary file 2

Supplementary file 1
