# Supplementary material for: Value added medicines: what value repurposed medicines might bring to society?
Source: J Mark Access Health Policy. 2016 Dec 23;5(1):1264717. doi: 10.1080/20016689.2017.1264717 (PMC5328340; doi:10.1080/20016689.2017.1264717)
Supplement: Supplementary file 3 [file zjma_a_1264717_sm7479.docx]

**Supplementary File 3-****Written survey dedictated to representatives of pharmaceutical industry developing value added medicines**

1. **Why some companies are engaging in the development of value added medicines?**
2. **Which companies are more engaged in value added medicines development? Please elaborate.**
3. Large generic companies
4. Small-medium generic companies
5. Large brand companies
6. Small-medium brand companies
7. **What are the 5 most important drivers and barriers to engage in the development of a specific value added medicines? Please elaborate.**
8. **Could you please list 3 or more countries more open to value added medicines and 3 or more countries refractory to value added medicines? Please elaborate.**
9. **Among these 3 steps, regulatory, HTA bodies, payers, which one do you consider as the most important hurdle for value added medicines? Please elaborate.**
10. **Please list 2 cases studies where:**
11. Value added medicines were not rewarded by HTA bodies
12. Value added medicines were rewarded by HTA bodies
13. **Please list 2 case studies where:**
14. HTA body recognition did not lead to a premium price
15. Absence of HTA body recognition led to premium price
16. **Do you have any general comments related to value added medicines?**
