# Supplementary material for: Value added medicines: what value repurposed medicines might bring to society?
Source: J Mark Access Health Policy. 2016 Dec 23;5(1):1264717. doi: 10.1080/20016689.2017.1264717 (PMC5328340; doi:10.1080/20016689.2017.1264717)
Supplement: Supplementary file 2 [file zjma_a_1264717_sm7442.docx]

**Supplementary File 2-** **Value added medicines examples presented during the European Patient’s Forum – Medicines for Europe Dialogue that took place on 31^st^ May 2016**

| **Case 1** | A company is developing a fixed-dose combination of 2 products already available on the market and used as free dose combination in arterial hypertension to reduce pill burden and avoid intake errors in a highly medicated patient population. |
| --- | --- |
| **Case 2** | A company is developing a self-injected subcutaneous formulation of a product already available on the market as intravenous formulation administered only at hospital under medical monitoring in a severe inflammatory disease. |
| **Case 3** | A company is developing a new formulation of a well-known chemotherapy product helping to reduce serious side effects of the original product used in many chemotherapy regimens. |
| **Case 4** | A company is re-positioning a well-known product in a rare pediatric indication as an alternative to reference treatments not specifically approved in this indication. |
| **Case 5** | A company is developing a new inhaled device to administer genericised products in COPD indication with evidence of reducing inhaler errors versus current device used with these active substances. |
| **Case 6** | A company is developing an extended-release formulation of a product already available on the market reducing administration regimen from once-weekly injection to 3-monthly injection in a neurocognitive disease indication. |
| **Case 7** | A company is developing a therapeutic drug monitoring device in association with a known cancer therapy exhibiting a narrow therapeutic window to optimize drug efficacy while minimizing toxicity. |
| **Case 8** | A company is developing an injectable biological to be kept refrigerated that will be provided to the patients with cool bags and sharp containers (not provided with the reference product) aiming to facilitate daily usage by the patients. |
