# Supplementary material for: Value added medicines: what value repurposed medicines might bring to society?
Source: J Mark Access Health Policy. 2016 Dec 23;5(1):1264717. doi: 10.1080/20016689.2017.1264717 (PMC5328340; doi:10.1080/20016689.2017.1264717)
Supplement: Supplementary file 1 [file zjma_a_1264717_sm7434.docx]

**Supplementary File 1-Discussion guide for value added medicines (audience: Healthcare providers/Payers/HTA bodies/Regulatory Authorities)**

1. **Healthcare system inefficiencies**
2. Do you see any healthcare system inefficiencies related to medicines? Please elaborate.
3. Do you see a need to improve existing medicines? Please elaborate.
4. **Value added medicine knowledge**
5. Are you familiar with value added medicines terminology? If yes, in which circumstances have you heard about value added medicines terminology?
6. Are you familiar with other terminologies underlying the same concept? If yes, which ones.
7. Would you have in mind some categories/examples of medicines that you would qualify as value added medicines?
8. What is your global perception of this category of medicines?
9. **Reaction to value added medicines profiles**

We present below 8 cases of products that might be qualified as value added medicines.

| **Case 1** | A company is developing a fixed-dose combination of 2 products already available on the market and used as free dose combination in arterial hypertension to reduce pill burden and avoid intake errors in a highly medicated patient population. |
| --- | --- |
| **Case 2** | A company is developing a self-injected subcutaneous formulation of a product already available on the market as intravenous formulation administered only at hospital under medical monitoring in a severe inflammatory disease. |
| **Case 3** | A company is developing a new formulation of a well-known chemotherapy product helping to reduce serious side effects of the original product used in many chemotherapy regimens. |
| **Case 4** | A company is re-positioning a well-known product in a rare pediatric indication as an alternative to reference treatments not specifically approved in this indication. |
| **Case 5** | A company is developing a new inhaled device to administer genericised products in COPD indication with evidence of reducing inhaler errors versus current device used with these active substances. |
| **Case 6** | A company is developing an extended-release formulation of a product already available on the market reducing administration regimen from once-weekly injection to 3-monthly injection in a neurocognitive disease indication. |
| **Case 7** | A company is developing a therapeutic drug monitoring device in association with a known cancer therapy exhibiting a narrow therapeutic window to potentialise drug efficacy while minimizing toxicity. |
| **Case 8** | A company is developing an injectable biosimilar to be kept refrigerated that will be provided to the patients with cool bags and sharp containers (not provided with the reference product) aiming to facilitate daily usage by the patients. |

1. Do you consider all of these products as equivalent in terms of added value for patients? Please elaborate.
   - 1. Would you value some of these cases at similar level than “a new chemical entity”? Please elaborate.
2. Beyond patient added-value, which impact would you foresee from society perspective with such therapies?
3. **Current value added medicine recognition**
4. From your experience, what are the main hurdles for value recognition of value added medicines in your country? Please elaborate.
5. From your experience, in which circumstances benefit of value added medicines would be acknowledged? Please elaborate.
6. Do you see any opportunity that value added medicines would help achieving your own interests or objectives? Please elaborate.
7. **Enhancement of VAM Recognition**
8. From your perspective do you consider any alternative or tailored pathway that might be implemented to enhance value added medicine recognition? Please elaborate.
9. Which arguments would you suggest to voice a better recognition of value added medicines?
10. Could you please comment on a new suggestion for value added medicine definition? Please comment.

*“Medicines based on known molecules which address unmet medical need and/or deliver relevant additional improvement for patients, health care professionals and/or payers.*

*The added value may be achieved through drug repositioning, drug reformulation, drug combination or new added service.*

*Relevant benefits will include better convenience of use, improved efficacy, safety and tolerability profile, better adherence, better quality of life, and/or patient preference.*

*These benefits are expected to impact health care systems in terms of reduction of healthcare use, budget impact, preventing therapeutic escalation, rationale use of medicines, improving equity ultimately contributing to address inefficiencies of the health care system.”*

1. **Conclusion**
2. What would be your recommendations for a company willing to engage in the development of VAM?
